# Isolation, genome analysis and comparison of a novel parainfluenza virus 5 from a Siberian tiger (*Panthera tigris*)

**DOI:** 10.3389/fvets.2024.1356378

**Published:** 2024-04-05

**Authors:** Niu Zhou, Liang Chen, Chen Wang, Mengna Lv, Fen Shan, Wanping Li, Yajiang Wu, Xueqing Du, Jinli Fan, Minting Liu, Menghan Shi, Jingjing Cao, Junqiong Zhai, Wu Chen

**Affiliations:** ^1^Guangzhou Zoo, Guangzhou, China; ^2^Guangzhou Wildlife Research Center, Guangzhou, China; ^3^Agriculture and Rural Bureau of Yuanzhou District, Yichun, China; ^4^Anan Pet Hospital, Suzhou, China; ^5^State Key Laboratory of Microbial Technology, Microbial Technology Institute, Shandong University, Qingdao, China

**Keywords:** Siberian tiger, parainfluenza virus 5, etiology, isolation, genome analysis, transmission electron microscopy, phylogenetic analysis, interspecies transmission

## Abstract

Paramyxoviruses are important pathogens affecting various animals, including mammals and humans. Parainfluenza virus 5 (PIV5)—a member of the family *Paramyxoviridae*—is a major threat to the health of mammals and humans. However, studies on terrestrial wild animals infected with PIV5 are scanty. In this study, we utilized reverse transcription PCR to detect PIV5 infection in the visceral organ tissues of a Siberian tiger (*Panthera tigris* ssp. *altaica*) with vomiting, diarrhea, and dyspnea before its death. A novel PIV5 (named SR strain) with a slowly progressive cytopathic effect was isolated in Vero cells and validated using a transmission electron microscope. Full-length sequencing and analysis revealed that the whole genome of the PIV5 SR strain contained 15,246 nucleotides (nt) and seven non-overlapping genes (3’-N-V/P-M-F-SH-HN-L-5′) encoding eight proteins. Phylogenetic analysis of three PIV5 strains identified in the same zoo confirmed that PIV5 strains SR and ZJQ-221 shared the closest genetic relationship as they were clustered in the same branch, while the recently found Siberian tiger strain SZ2 kept a certain distance and formed a relatively unique branch. Furthermore, mutations of nt and amino acids (aa) between strains ZJQ-221, SR, and SZ2 were identified. In summary, we report the identification and genomic characterization of a novel PIV5 strain SR isolated in a Siberian tiger, which may help future research on interspecific transmission mechanisms.

## Highlights

- A novel PIV5 strain (named SR) was isolated from a Siberian tiger (*Panthera tigris* ssp. *altaica*) with clinical symptoms.
- PIV5 SR strain infection was diagnosed by molecular biology and caused a slowly progressive cytopathic effect in Vero cells, the virions of which were imaged using a transmission electron microscope.
- Full-length sequencing and analysis of the PIV5 SR strain genome were performed for alignment and phylogenesis.
- Mutations of nucleotides and predicted viral proteins were found in ZJQ-221, SR, and SZ2 isolated from the same zoo, which might help to explore the potential pattern of evolution and interspecies transmission.

## Background

Parainfluenza virus 5 (PIV5)—originally known as canine parainfluenza virus (CPIV) and simian virus 5 (SV5) for its first identification in primary monkey kidney cells in 1956 (1)—is a member of the genus *Rubulavirus* in the family *Paramyxoviridae* (2, 3). With a virion diameter of 50–200 nm, PIV5 has a non-segmented negative-sense single-stranded RNA of ~15,246 nucleotides (nt), which contains seven genes and encodes eight proteins (NP, V/P, M, F, SH, HN, and L) (4–7). The V and P proteins of PIV5 share the same genomic encoding region and are encoded by a specific RNA editing mechanism (8).

*Paramyxoviruses* (PVs) represent important zoonotic pathogens with implications for the central nervous system, encephalitis, and respiratory systems, posing risks for both animal and human health (9–12). They are members of the *Paramyxoviridae* family and exhibit a global prevalence in various animal populations, including PIV5 in Korean porcine (13) and in Switzerland cattle populations (9). In addition, peste des petits ruminant viruses have been identified in the Comoros Archipelago (14), canine distemper virus (CDV) in captive Siberian tigers and red pandas (15), and novel PVs of the *Jeilongvirus* genus in bats from China (16). Notably, Nipah and Hendra viruses were predicted to have the potential to cause the next zoonotic pandemic (10). Zoonotic pathogens, such as PVs, have demonstrated the ability to cross species boundaries, leading to zoonotic outbreaks and posing a public health risk over the past two decades. Particularly, PVs exhibit a wide range of common hosts, suggesting a heightened potential for interspecies transmission. The role of the tick as an intermediate host has been proposed, promoting the transmission of PVs among mammals (17). Nonetheless, the evolutionary dynamics of PVs during interspecies transmission remain inadequately studied. This knowledge gap underscores the need for further research to enhance our understanding of the mechanisms underlying the evolution of PVs in the context of interspecies transmission.

To investigate the etiology of the death of the Siberian tiger (*Panthera tigris* ssp. *altaica*) exhibiting symptoms of vomiting, diarrhea, and dyspnea, we utilized RT-PCR, virus isolation, and electron microscopy, ultimately confirming PIV5 infection. Subsequently, we conducted phylogenetic and evolutionary analyzes and compared nt and amino acid (aa) mutations of the successive PIV5 strains SR, SZ2, and ZJQ-221 identified in the zoo. These findings might provide valuable insights into the prevalence and the interspecies transmission mechanisms of PIV5.

## Materials and methods

### Samples

A 12-year-old male Siberian tiger (~230 kg) died in 2015 at a zoo in Guangdong province in southern China after vomiting, diarrhea, and dyspnea for approximately a month. Lobular pneumonia was observed after necropsy, and tissue samples of the livers, spleen, lungs, and kidneys were collected and stored at −80°C.

### Reverse transcription PCR (RT-PCR)

Tissue samples of the Siberian tiger were tested for the possible presence of PIV5, feline parvovirus (FPV), feline coronavirus (FCoV), and CDV using RT-PCR according to previous studies (18). Total RNA of tissue homogenates was extracted using a RNeasy Lipid Tissue Kit (Qiagen, CA, United States) and reverse transcribed using the Transcriptor First Strand cDNA Synthesis Kit (Roche Diagnostics, Mannheim, Germany) according to the manufacturers’ recommendations. PCR assays were carried out to detect viral nt using a pair of detection primers named PIV5-1-F/R, and full-length genome sequences were amplified using a set of 12 pairs of primers following our previous study (19). Complementary DNA (cDNA) samples as templates were added to a total volume of 25 μL using PrimeSTAR^®^ Max DNA Polymerase (TaKaRa, Japan) according to the manufacturer’s protocol. PCR reactions were performed using the following conditions: 95°C for 4 min; 35 cycles of 95°C for 30 s, 55°C for 30 s, and 72°C for 45 s to 2 min; and final extension of 72°C for 10 min.

### Gel electrophoresis, sequencing, and similarity analysis

To separate the indicated DNA fragments, the prepared PCR products were added to a 1% agarose gel with Golden View™ (TaKaRa) and electrophoresed with DL2000 DNA marker (TaKaRa) at 120 V for approximately 25 min, as we reported (20). PCR band products were visualized using a Gel Doc™ EZ imaging system (Bio-Rad, CA). Positive PCR products were purified (MiniBEST Agarose Gel DNA Extraction Kit, TaKaRa), tailed with “A”-overhang tails (DNA A-Tailing Kit, TaKaRa), and cloned into the pMD19-T vector (TaKaRa). Positive recombinant plasmids were sequenced by Ruibiotech Co., Ltd. (Beijing, China). Nucleotide sequences were blasted against sequences deposited in GenBank using the basic local alignment search tool, nt (BLASTn)^1^ for similarity analysis.

### Virus isolation

Tissue samples were homogenized in sterile phosphate-buffered saline (PBS, pH 7.4) in a ratio of 1/10 (w/v) and centrifuged at 5,000 × g for 10 min at 4°C. Then, supernatants filtered with 0.22-μm membrane were inoculated onto 90% monolayer Vero cells with Dulbecco’s minimal essential medium (DMEM, Gibco) and incubated at 37°C under 5% CO_2_ for 1 h. Cells were then maintained in DMEM containing 3% fetal bovine serum (FBS, Gibco) and 1% Pen-Strep (21, 22) at 37°C under 5% CO_2_ and monitored daily for the cytopathic effect (CPE). Cell supernatants were collected after a freeze–thawing cycle three times when the CPE reached ~70% and stored at −80°C as virus stock.

### TEM scanning

To observe virus particles in infected cells, cytopathic Vero cells infected with virus stock were scraped gently and fixed using 2.5% glutaraldehyde fixation fluid at 4°C for 4 h. Finally, ultrathin sections of the infected cell aggregate samples were stained with 2% uranium acetate and 2.6% lead citrate and then scanned on a Hitachi TEM system (Hitachi H-7000FA, Japan).

### Sequence alignment and phylogenetic analysis

Nucleotide sequence alignment based on different rubulavirus species genome sequences was performed using DNAStar Lasergene 7.10 (Madison, WI, United States), and phylogenetic analysis was performed using MEGA 7 (23) with the maximum likelihood algorithm. Bootstrap values were calculated with 1,000 replicates. Geneious Prime (Version 2021.1.1) was used to compare sequences of nt and related encoding proteins of PIV5 SR, ZJQ-221, and SZ2 strains isolated in the same zoo, and then the results of nt and aa mutations were organized using Adobe Photoshop CC 2019.

## Results

### Isolation and identification of PIV5 from Siberian tiger

**Figure 1:**
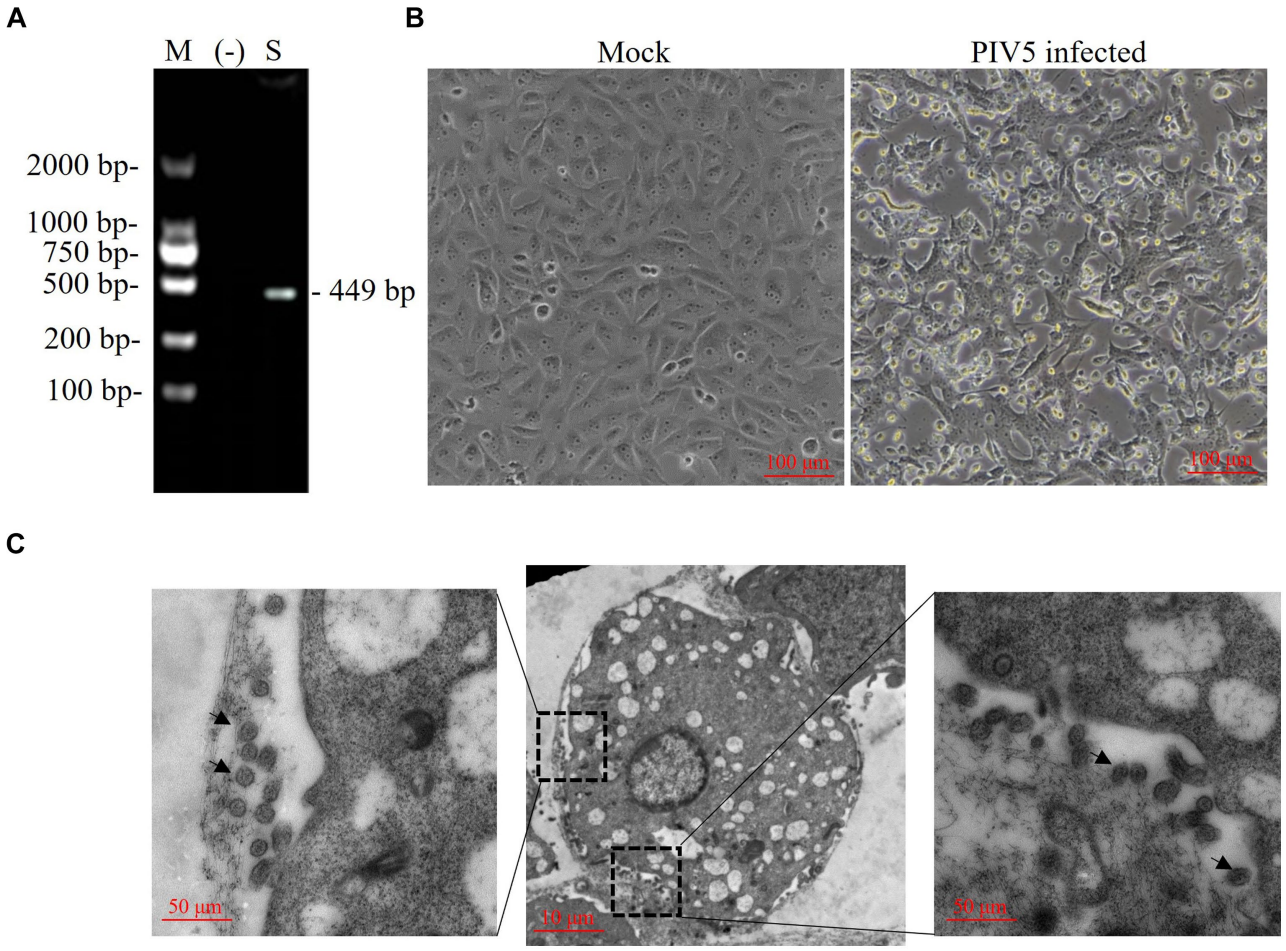
Identification of a paramyxovirus isolated from a Siberian tiger. **(A)** PIV5 viral nucleotides (nt) were detected in a Siberian tiger sample using reverse transcription PCR (RT-PCR). M, DL2000 DNA marker; −, negative control; S, Siberian tiger sample. **(B)** Vero cells of mock infection (left) and PIV5 infection (right) using an inverted microscope. Bar, 100 μm. **(C)** Transmission electron microscopy images of Vero cells inoculated with PIV5 virus stock. Bar, 10 μm or 50 μm; black arrow, viral particles.

A Siberian tiger in a zoo of southern China died after vomiting, diarrhea, and dyspnea, and tissue samples of which were collected during necropsy and tested for virus infection. RT-PCR revealed the positive presence of PIV5 nt in the Siberian tiger tissue samples (Figure 1A), which were negative for FCoV, FPV, or CDV (data not shown). The supernatants of tissue homogenates were prepared for virus isolation by inoculating Vero cells with a 90% monolayer for several passages. Compared with uninfected cells, Vero cells inoculated with virus stocks from tissue homogenates for 5 days post-infection (dpi) showed loose attachment, roundness, and random orientation, which indicated a slowly progressive CPE (Figure 1B) of this PIV5 strain. PIV5 infection in Vero cells was further confirmed by TEM, which exhibited spherical particles with a diameter of 50–200 nm (Figure 1C). Thus, the PIV5 strain from the Siberian tiger was isolated and named SR.

### Genome characterization of PIV5 SR strain

**Figure 2:**
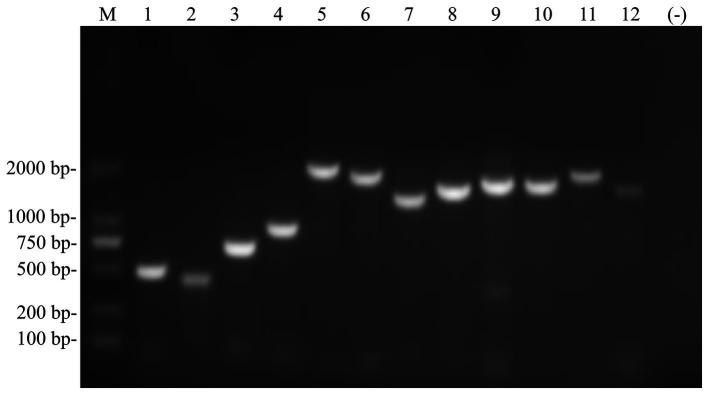
Full-length genome sequence of the PIV5 SR strain was amplified using RT-PCR. M, DL2000 DNA Marker; 1–12, 12 pairs of primers were used to amplify the full-length of the PIV5 SR strain.

A set of 12 pairs of primers was designed to amplify the full-length amplification of genome sequences from Siberian tiger tissue samples (Figure 2) as previously reported. After sequencing, alignment, and high similarity analysis, a full genome of the PIV5 SR strain with 15,246 nt was obtained, encompassing a 3′ leader sequence (55 nt), a non-overlapping encoding area (15,160 nt), and a 5′ trailer sequence (31 nt). The encoding area was predicted to encode seven viral proteins, including the NP gene (position: 152–1,681), V/P gene (position: 1,850–2,518), M gene (position: 3,141–4,274), F gene (position: 4,530–6,185), SH gene (position: 6,303–6,437), HN gene (position: 6,584–8,281), and L gene (position: 8,414–15,181), and six non-coding interval sequences between each gene. The complete genome sequence of the PIV5 SR strain was deposited in GenBank under the accession number KY685075.

### Sequence alignments and phylogenetic analysis of PIV5 SR strain

**Figure 3:**
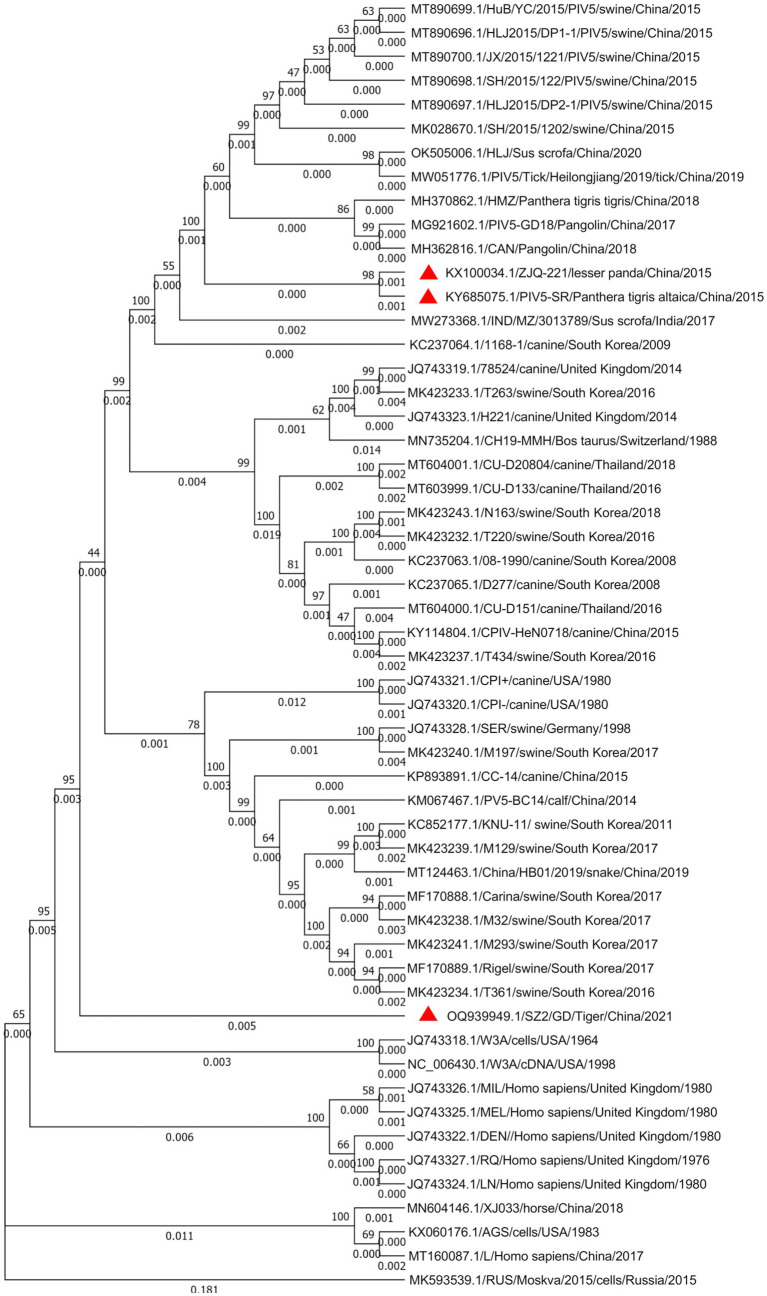
Phylogenetic analysis of the PIV5 SR strain based on the nucleotide sequences of the complete viral genome sequence. The nucleotide sequences of various parainfluenza virus strains were aligned using MEGA 7. One thousand bootstrap replicates were subjected to nt sequence distance and neighbor-joining analyzes, and a consensus phylogenetic tree was generated. Red triangles indicate strains found in the same zoo.

Whole-genome sequence alignment showed that the PIV5 SR strain had the lowest nt similarity (~97.13%) with the D277 strain of canine-origin (GenBank: KC237065) from South Korea and the highest nt similarity (~99.76%) with the ZJQ-221 strain of lesser panda (GenBank: KX100034) isolated from the same zoo. Phylogenetic analysis further confirmed the close genetic relationship between SR and ZJQ-221, as they were clustered in the same branch (Figure 3). Interestingly, the PIV5 SZ2 strain (GenBank: OQ939949.1), recently isolated from another dead Siberian tiger in the same zoo using Metavirome sequencing, kept a distance from the branch of SR and ZJQ-221 strains and formed an individual branch, indicating that SZ2 might have been evolved in a different way.

### Nt and aa comparisons of PIV5 ZJQ-221, SR, and SZ2 strains

**Figure 4:**
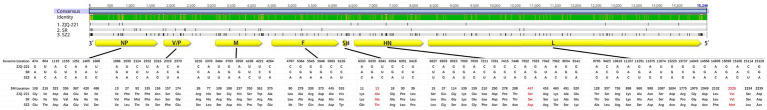
Sense mutations of lesser panda PIV5 strain ZJQ-221 and Siberian tiger PIV5 strains SR and SZ2 identified from the same zoo. Amino acid (aa) sequences of ZJQ-221, SR, and SZ2 strains were analyzed using Geneious Prime (Version 2021.1.1). The schematic diagram of the PIV5 genome structure and aa mutations was illustrated. Aa sense mutations resulting from two nt site mutations in one codon are shown in red. Yellow box, viral protein encoding area and direction in the genome. NP, nucleocapsid protein. V/P, V protein/phosphoprotein. M, Matrix protein. F, fusion protein. SH, small hydrophobic protein. HN, hemagglutinin-neuraminidase protein. L, large protein, or RNA-dependent RNA polymerase.

Since Siberian tiger strains SR and SZ2, as well as lesser panda strain ZJQ-221, were all found in the same zoo, these three strains might be helpful in exploring the potential pattern of evolution and interspecies transmission of PIV5. Thus, ZJQ-221, SR, and SZ2 sequences were analyzed using Geneious Prime (Version 2021.1.1), and mutation sites of nt and aa between strains are listed in Supplementary Table S1. The three PIV5 strains had several sense mutations in viral encoding areas (Figure 4). Intriguingly, no sense mutation in F or SH was found between ZJQ-221 and SR, while the newly found PIV5 strain SZ2 had 6 and 5 sense mutations in each viral protein, respectively. Furthermore, compared with ZJQ-221 and SR strains, three aa mutations of the PIV5 SZ2 strain in SH (13: Ala to Thr), HN (447: Ser to Asp), and L (2,229: Val to Met) resulted from two nt mutations in each codon (Figure 4).

## Discussion

The *Paramyxoviridae* family boasts a broad spectrum of viral reservoirs and is implicated in various diseases, including mumps and measles in humans, Newcastle disease in poultry, and distemper in carnivorous animals (24–26). Since its identification in 1954, PIV5 has evolved into a globally infectious agent over the past half century. Research has revealed that PIV5 can infect a diverse array of mammals, including humans, dogs, pigs, rats, rabbits, foxes, and cats, across different countries and regions. Despite the extensive knowledge of the infectivity of PIV5 in various species, the evolutionary dynamics of PVs during interspecies transmission remain elusive. While the virus has demonstrated its ability to traverse species boundaries, the mechanisms and factors influencing its evolution in the context of interspecies transmission are yet to be fully elucidated. This knowledge gap underscores the need for further investigations to unravel the intricacies of the evolutionary pathways of PVs during interspecies transmission.

In this study, a novel PIV5 strain named SR, with a full length of 15,246 nt and exhibiting spherical particles with a diameter of 50–200 nm consistent with previously reported studies (7, 13), was isolated from a captive Siberian tiger with clinical symptoms. The isolated PIV5 SR strain conforms to Koch’s postulates (Supplementary Table S1). Meanwhile, alignment and high similarity analysis demonstrated that this novel PIV5 SR strain shared the same genome structure and had the highest nt similarity (~99.76%) with the ZJQ-221 strain of lesser panda (GenBank: KX100034) from the same zoo. Additionally, the PIV5 SR strain was predicted to encode a small hydrophobic (SH) protein, which is a type II membrane protein of 44 aa residues, including a 5-aa C-terminal ectodomain, a 23-aa transmembrane domain, and a 16-aa N-terminal cytoplasmic region (13, 27). The SH protein was absent in PIV5 strains isolated from pigs, dogs, calves, and cells (19), implying that this protein might be dispensable in the lifecycle of PVs (28–31). However, the absence of SH protein in PIV5 induced apoptosis of infected cells by activating tumor necrosis factor-alpha expression (32). Interestingly, the role of SH protein in the inhibition of apoptosis, which contributes to the virulence of PV members, was further confirmed in the J paramyxovirus (JPV), mumps virus, and respiratory syncytial virus (RSV) (33). Our study identified the presence of the SH protein in tiger PIV5 strains SR and SZ2. The presence of this protein is speculated to play a role in reducing apoptosis and contributing to virulence throughout the lifecycles of these strains, thereby facilitating transmission among diverse host species.

One specific mutation in proteins might play an important role in the lifecycle, pathogenesis, evolution, and even interspecies transmission of viruses (34). It has been previously reported that mutations in the F fusogenic peptide (G3A) and near the F transmembrane domain (S443P) not only enhanced viral fusion activity but also increased viral susceptibility to antibody-mediated neutralization (35). A point mutation of glutamine at position 202 of the RNA-binding domain of human parainfluenza virus type 2 (hPIV2) nucleocapsid protein (NP) enhanced polymerase activity by approximately 30-fold, whereas a recombinant hPIV2 possessing the NP Q202A mutation could not be recovered from cDNA (36). Moreover, leucine at position 302 of the M protein of hPIV3 played a crucial role in viral RNA synthesis by regulating inclusion body formation (12). Hemagglutinin-neuraminidase (HN) of PV is a multifunctional protein mediating hemagglutination, neuraminidase, and fusion promotion activities. A study of PIV5 HN ectodomain structure revealed that V81T and L85Q mutations in the stalk region significantly impaired cell–cell fusion, while the D398L mutation within the head domain showed reduced fusion activity (37). A second receptor binding site on hPIV3 HN contributed to the activation of the fusion mechanism during host cell invasion (38). However, the creation of a second receptor binding site by site-specific mutagenesis at residue 523 on hPIV1 HN did not significantly affect the growth or fusion activity of the recombinant virus (39). pH-dependent (acid-activated) channel activity of human RSV SH proteins in transiently expressing HEK 293 cells was abolished when both His^22^ and His^51^ residues of the SH protein were mutated, but not when either was present (40). An additional mutation in E1658D of the PIV5 L protein might enhance virus replication in Vero cells when PIV5 without the conserved C-terminal of the V protein was inserted with hemagglutinin from the H5N1 Influenza A virus between the HN and L genes in the genome (41). Post-translation modification of specific residues of viral proteins also plays a vital role in virus transmission in hosts. The phosphoprotein status of PIV5 viral phosphoprotein (P) acted as a replication switch during virus replication (42), while SUMOylation played a key role in the growth of PIV5. Mutation of the P protein at 254 lysine to arginine (K254R) reduced PIV5 minigenome activity and the SUMOylation level of the P protein (43). The present study compared nt and protein mutations, which may help in exploring mechanisms underlying the evolution and interspecies transmission of PIV5.

In the current study, a novel PIV5 strain, designated SR strain, causing slowly progressive CPEs in Vero cells was isolated from a dead Siberian tiger with clinical symptoms including vomiting, diarrhea, and dyspnea. Virions of the PIV5 SR strain in infected cells were imaged using a transmission electron microscope (TEM). The full genome of the SR strain showed a classical PIV5 genome structure characteristic and the closest genetic relationship with a lesser panda strain, ZJQ-221, isolated in the same zoo. Furthermore, mutations of nt and aa in SR, SZ2, and ZJQ-221 strains were identified. Our study findings provide insight into the epidemiology and genomics of PIV5 and highlight the urgent need to control PIV5 in zoo animals to avoid interspecies transmission. The occurrence of PIV5 mutations in these wild animals might provide potential candidates for future research on the molecular mechanisms underlying virus evolution and interspecies transmission.

## Data availability statement

The datasets presented in this study can be found in online repositories. The names of the repository/repositories and accession number(s) can be found in the article/Supplementary material. The data presented in the study are deposited in the NCBI repository, SRA accession number: SRR27458479.

## Ethics statement

The animal study was approved by Guangzhou Zoo Ethics Committee. The study was conducted in accordance with the local legislation and institutional requirements.

## Author contributions

NZ: Project administration, Writing – original draft, Writing – review & editing. LC: Project administration, Writing – original draft. CW: Data curation, Formal analysis, Writing – original draft. MeL: Project administration, Writing – original draft. FS: Investigation, Methodology, Writing – original draft. WL: Methodology, Project administration, Writing – original draft. YW: Investigation, Methodology, Writing – original draft. XD: Methodology, Writing – original draft. JF: Data curation, Writing – original draft. MiL: Data curation, Writing – original draft. MS: Methodology, Writing – original draft. JC: Data curation, Writing – original draft, Writing – review & editing. JZ: Writing – original draft, Writing – review & editing. WC: Writing – original draft, Writing – review & editing.

## Footnotes

^1^https://blast.ncbi.nlm.nih.gov/Blast.cgi
