# Supplementary material for: Isolation, genome analysis and comparison of a novel parainfluenza virus 5 from a Siberian tiger (Panthera tigris)
Source: Front Vet Sci. 2024 Apr 5;11:1356378. doi: 10.3389/fvets.2024.1356378 (PMC11057237; doi:10.3389/fvets.2024.1356378)
Supplement: Supplementary file 1 [file Image_1.pdf]

Supplementary Figure Legends

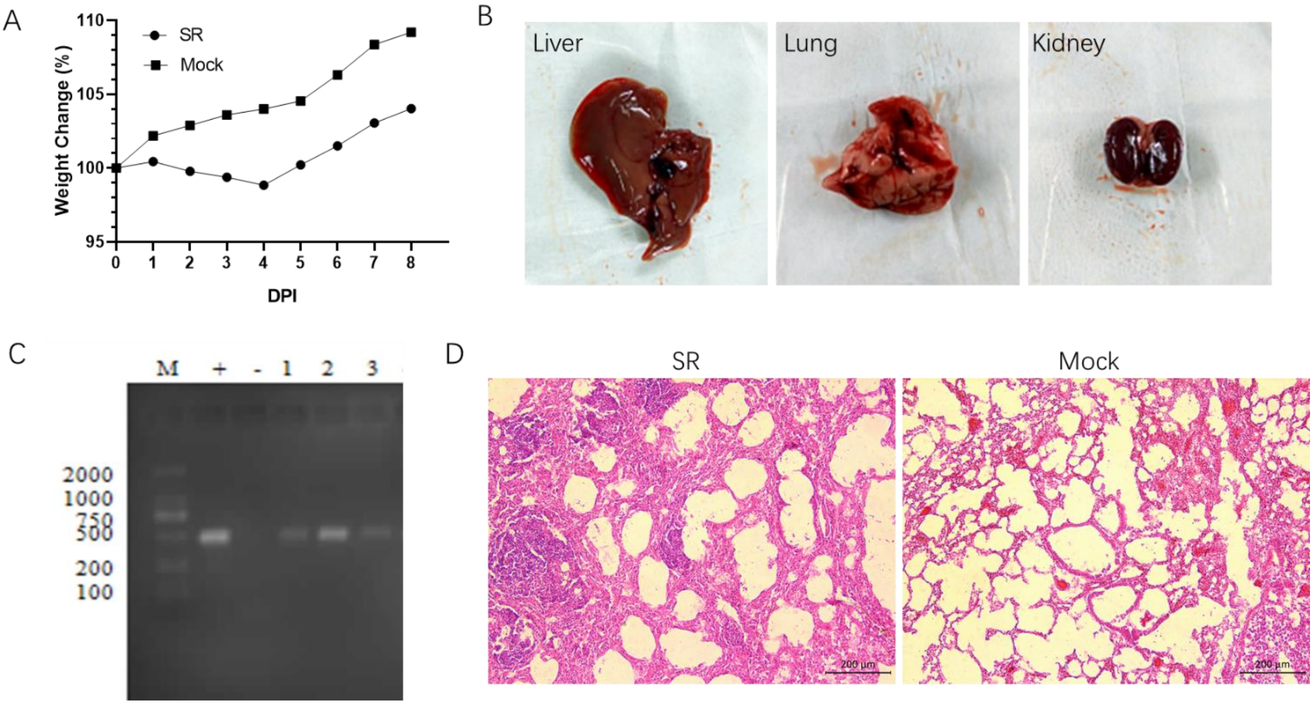

Supplementary Figure 1: Specific pathogen-free Kunming mice were infected with 50 μl of PIV5 SR strain virus stock (SR group) or inoculated with Dulbecco's minimal essential medium (DMEM) (Mock group) via intranasal administration and observed for 8 days. (A) Mouse weights were measured daily and calculated to determine weight changes. (B) Eight days post-infection (DPI), the livers (left), lungs (middle) and kidneys (right) of the mice from the SR group were analyzed. Compared with the Mock group, the lungs of the SR group showed gross lesions of consolidation and hemorrhagic spots. (C) RT-PCR detection results of PIV5 showed liver, lung and kidney in SR group were all positive for PIV5. 1-3, represented the liver, lung and kidney of the SR group. +, positive control. -, negative control. M, DNA ladder. (D) Hematoxylin & eosin staining of lungs of mice from SR and Mock groups. The results showed that mice in the SR group had pneumonia with thickened alveolar walls and a small amount of exuding inflammatory cells.
